# Identification and characterization of archaeal-type FAD synthase as a novel tractable drug target from the parasitic protozoa *Entamoeba histolytica*

**DOI:** 10.1128/msphere.00347-24

**Published:** 2024-08-27

**Authors:** Dewi Wulansari, Ghulam Jeelani, Euki Yazaki, Tomoyoshi Nozaki

**Affiliations:** 1 Department of Biomedical Chemistry, Graduate School of Medicine, The University of Tokyo, Tokyo, Japan; 2 National Research and Innovation Agency, Jakarta, Indonesia; 3 Research Center for Advanced Analysis, National Agriculture and Food Research Organization, Ibaraki, Japan; University of California, Davis, California, USA

**Keywords:** *Entamoeba histolytica*, protozoan, flavin adenine dinucleotide, FAD synthase, cofactor, riboflavin kinase, gene silencing, drug target

## Abstract

Flavin adenine dinucleotide (FAD) is an essential cofactor for numerous flavoenzymes present in all living organisms. The biosynthesis of FAD from riboflavin involves two sequential reactions catalyzed by riboflavin kinase and flavin adenine dinucleotide synthase (FADS). *Entamoeba histolytica*, the protozoan parasite responsible for amebiasis, apparently lacks a gene encoding FADS that share similarity with bacterial and eukaryotic canonical FADS, yet it can synthesize FAD. In this study, we have identified the gene responsible for FADS and thoroughly characterized physiological and biochemical properties of FADS from *E. histolytica*. Phylogenetic analysis revealed that the gene was likely laterally transferred from archaea. The kinetic properties of recombinant EhFADS were consistent with the notion that EhFADS is of archaeal origin, exhibiting *K*_M_ and *k*_cat_ values similar to those of the arachaeal enzyme while significantly differing from the human counterpart. Repression of gene expression of *EhFADS* by epigenetic gene silencing caused substantial reduction in FAD levels and parasite growth, underscoring the importance of EhFADS for the parasite. Furthermore, we demonstrated that *EhFADS* gene silencing reduced thioredoxin reductase activity, which requires FAD as a cofactor and makes the ameba more susceptible to metronidazole. In summary, this study unveils unique evolutionary and biochemical features of EhFADS and underscores its significance as a promising drug target in combating human amebiasis.

**IMPORTANCE:**

FAD is important for all forms of life, yet its role and metabolism are still poorly studied in *E. histolytica*, the protozoan parasite causing human amebiasis. Our study uncovers the evolutionary unique key enzyme, archaeal-type FADS for FAD biosynthesis from *E. histolytica* for the first time. Additionally, we showed the essentiality of this enzyme for parasite survival, highlighting its potential as target for drug development against *E. histolytica* infections.

## INTRODUCTION

Flavin adenine dinucleotide (FAD) is indispensable for all living organisms, serving as a cofactor for numerous flavin-dependent enzymes involved in diverse biological processes. These processes range from redox reactions, homeostasis, and energy metabolisms to protein folding, chromatin remodeling, DNA repair, apoptosis, and the biosynthesis of various secondary metabolites (1). Approximately 75% of flavin-dependent enzymes require FAD as a cofactor with the remaining 25% utilizing flavin mononucleotide (FMN) (2). Several FAD-dependent enzymes play vital roles in cell survival and have been explored as potential drug targets against bacteria, parasitic protozoa, and helminths. Examples include enzymes such as glutathione reductase (GR) (3, 4), thioredoxin reductase (TrxR) (5), thioredoxin glutathione reductase (6–8), and trypanothione reductase (9), which play crucial roles in cellular redox regulation. Malate quinone oxidoreductase (10, 11) and uridine diphosphate-galactopyranose mutase (12) are other FAD-dependent enzymes considered as promising targets for drug development.

**Fig 1:**
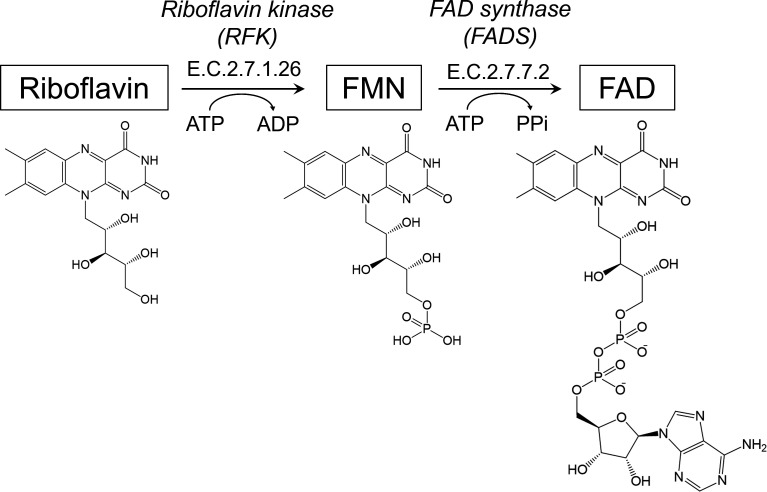
Schematic diagram of riboflavin metabolism. Riboflavin is converted to FMN by RFK and subsequently to FAD by FADS. FADS, flavin adenine dinucleotide synthase; PPi, pyrophosphate; RFK, riboflavin kinase.

FAD is the final product of riboflavin metabolism, involving two consecutive reactions. Riboflavin is first metabolized into FMN by the action of riboflavin kinase (RFK) (E.C.2.7.1.26) and subsequently into FAD by the action of FMN: ATP adenylyltransferase (FMNAT) (E.C.2.7.7.2) (13) (Fig. 1). In most prokaryotes, both reactions are catalyzed by one bifunctional enzyme, with riboflavin kinase activity in the C-terminal region and FMNAT activity in the N-terminal region (14). In contrast, eukaryotes perform these functions with two different proteins, RFK and flavin adenine dinucleotide synthase (FADS). While RFK is relatively well conserved in sequence and structure across eukaryotes and bacteria, FADS responsible for FMNAT activity largely varies between bacteria and eukaryotes. Bacterial FMNAT belongs to nucleotidylyl transferase superfamily with the conserved (H/T)xGH signature motif and ISSTxxR motif, which interact with the β- and γ-phosphates of the ATP nucleotide (15). Conversely, eukaryotic FADS belongs to 3-phosphoadenosine 5-phosphosulfate (PAPS) reductase-like family of the “adenine nucleotide α hydrolase-like” superfamily, sharing motifs for nucleotide binding and arginine residues forming an arginine grip (16). These arginine residues form a salt bridge either with the phosphate of ATP or FMN, while in PAPS reductase, the corresponding arginines form a salt bridge to the phosphosulfate of (P)APS (16).

*Entamoeba histolytica* is a protozoan parasite responsible for amebic dysentery, diarrhea, and extraintestinal amebiasis, resulting in about 100,000 deaths annually (17). Metronidazole and related 5-nitroimidazole compounds are currently the drug of choice for amebiasis. However, several concerns have been raised, including side effects, teratogenicity, and lack of efficacy against cyst (18). Moreover, metronidazole resistance in *E. histolytica* was documented *in vitro* and in clinical cases of other metabolically similar bacterial and protozoan pathogens, which share anaerobic metabolism. Thus, there are justifiable needs to identify novel targets against the parasite (19, 20). As in many other eukaryotic organisms, *E. histolytica* relies on extracellular riboflavin from the environment since the genes encoding riboflavin biosynthetic enzymes are absent from its genome (21). The essentiality of riboflavin was previously demonstrated by the growth inhibition caused by riboflavin deprivation from the culture medium, which can be restored by the addition of riboflavin (22). It was previously shown that *E. histolytica* can metabolize riboflavin into FMN and FAD (23), implying the presence of both RFK and FMNAT activities. However, only a gene encoding RFK, composed of 131 amino acids containing only the flavokinase domain, is identified in the *E. histolytica* genome, whereas a gene encoding a protein that possesses the FMNAT domain is not annotated in the genome. This study aims to identify FADS responsible for the final step of FAD biosynthesis in *E. histolytica* and to characterize physiological and biochemical properties of FADS to propose as a new rational drug target.

## RESULTS

### Identification and unique features of archaeal-type FADS from *E. histolytica*

Since neither a gene encoding FMNAT domain of bacterial-type RFK/FMNAT fusion protein nor a gene encoding an eukaryotic-type FADS was present in the *E. histolytica* genome database, AmoebaDB (https://amoebadb.org/amoeba/app), we hypothesized that *E. histolytica* may possess archaeal-type FADS gained by lateral gene transfer. To search for a FADS ortholog in *E. histolytica*, the amino acid sequence of RibL (24), an archaeal FADS of *Methanocaldococcus jannaschii* (NCBI accession no. WP_010870692), which has no sequence similarity with either prokaryotic or eukaryotic FADS, was used as a query sequence for BLASTP search against the *E. histolytica* genome. The best hit was a gene annotated as cytidylyltransferase, putative (gene ID: EHI_198800), sharing 41% identity with the query sequence (*E* value: 2e-23). The gene has no intron and contains a 507-bp open reading frame, encoding a protein of 168 a.a. with an estimated molecular weight of 19.4 kDa.

**Fig 2:**
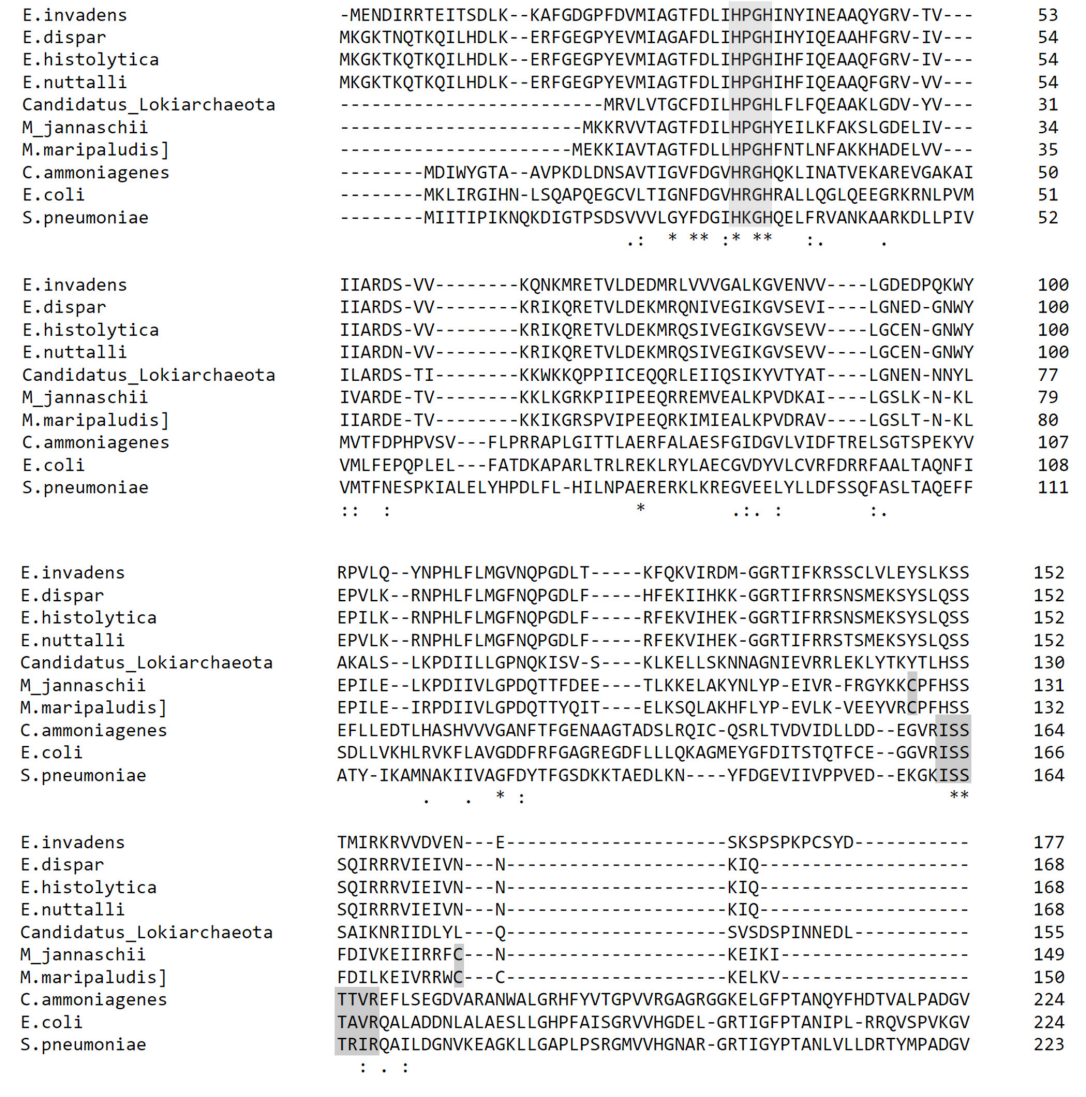
Protein alignment of EhFADS with its *Entamoeba* orthologs and bacterial FMNAT. Multiple alignment is made by Clustal O (25). Highlighted in gray are the conserved HxGH motif of nucleotidyltransferase, ISSTRxxR motif of bacterial FMNAT, and two cysteine residues present in archaea FADS, RibL. NCBI accession numbers for each species are XP_654999.1 (*E. histolytica*), XP_004261251.1 (*Entamoeba invadens*), XP_001738466.1 (*Entamoeba dispar*), XP_008856984.1 (*Entamoeba nuttalli*), MCF2141313.1 (*Candidatus Lokiarchaeota*), WP_010870692.1 (*M. jannaschii*), WP_011976597.1 (*Methanococcus maripaludis*), WP_258747795.1 (*Escherichia coli*), WP_061756253.1 (*Streptococcus pneumoniae*), and BAA07182.1 (*Corynebacterium ammoniagenes*).

**Fig 3:**
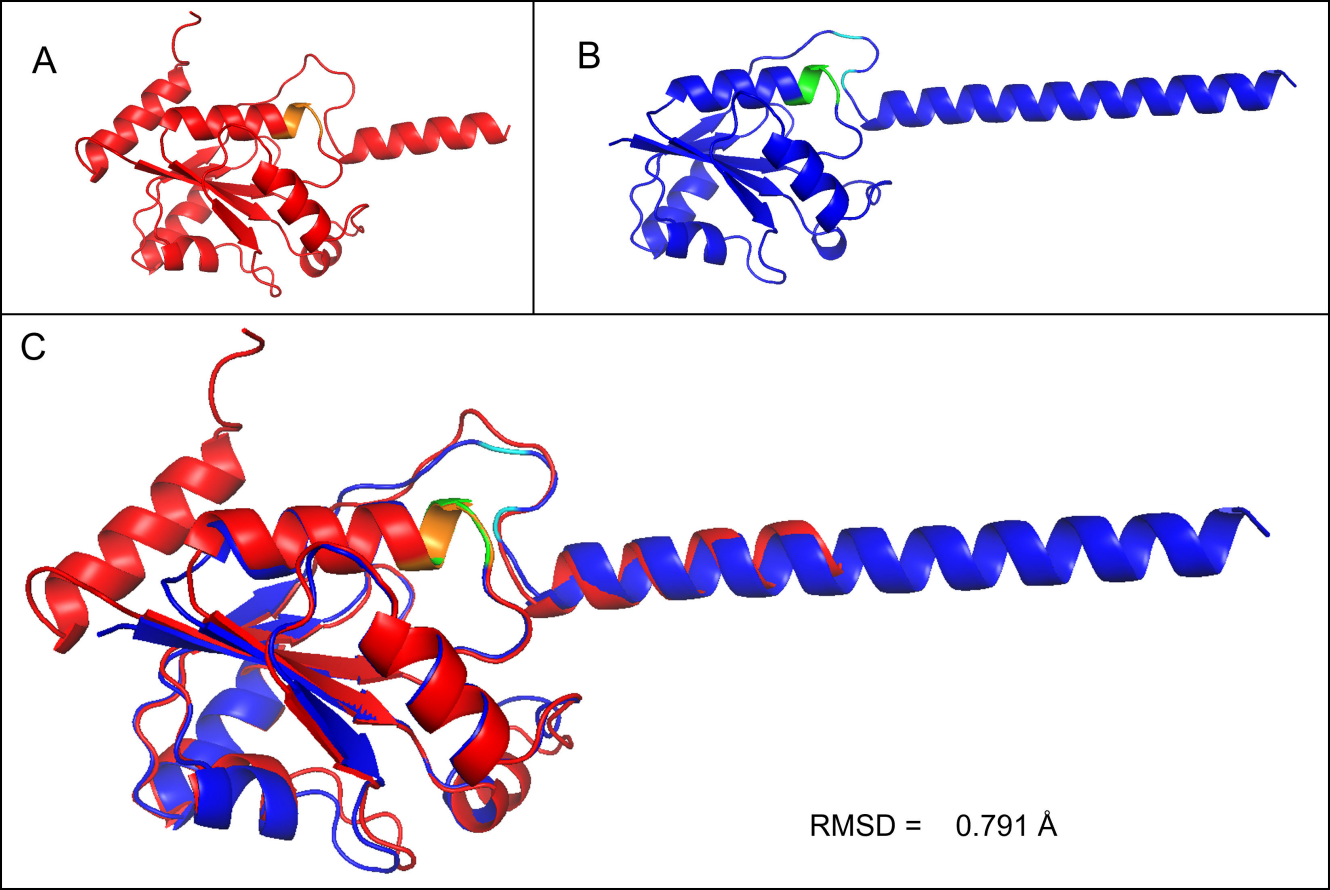
AlphaFold predicted structure of EhFADS and archaea FADS RibL. The predicted structure of EhFADS (A, AF-C4LWS6-F1, red) compared with RibL (B, AF-F2KSG9-F1, blue). (**C**) Alignment of EhFADS and RibL using PyMOL (The PyMOL Molecular Graphics System, version 1.7.4; Schrödinger, LLC). The conserved motif HxGH of EhFADS (orange) fits well with those of RibL (green). The two cysteine residues present in RibL are in cyan. RMSD, root mean square deviation.

Domain analysis of the protein sequence using Interproscan predicted that the protein is a member of the nucleotidylyltransferase superfamily (*E* value: 4.6e-18), like those of the FMNAT domain of bacterial FADS, and contains a cytidyltransferase-like domain which spans 28–158 a.a. Alignment of EHI_198800 with its ortholog from *Entamoeba* and possible orthologs of archaeal and bacterial origin showed the presence of conserved HxGH motif; however, ISSSTxxR is degenerated in EHI_198800 (Fig. 2). Both motifs are conserved in the nucleotidylyl transferase superfamily and known to be responsible for interaction with the β- and γ-phosphates of the ATP (15). AlphaFold prediction structure of EHI_198800 shows a similar structure to archaeal FADS, RibL, with a root mean square deviation of 0.791 Å (Fig. 3). The HxGH motif of both proteins fit well with each other. Unlike the archaeal RibL, which is air sensitive due to the presence of two cysteine residues (24), EHI_198800 does not contain any of those residues, suggesting that EHI_198800, if this is FADS, is not air sensitive, and no iron may be involved in the reaction.

### Phylogenetic analysis of EhFADS

**Fig 4:**
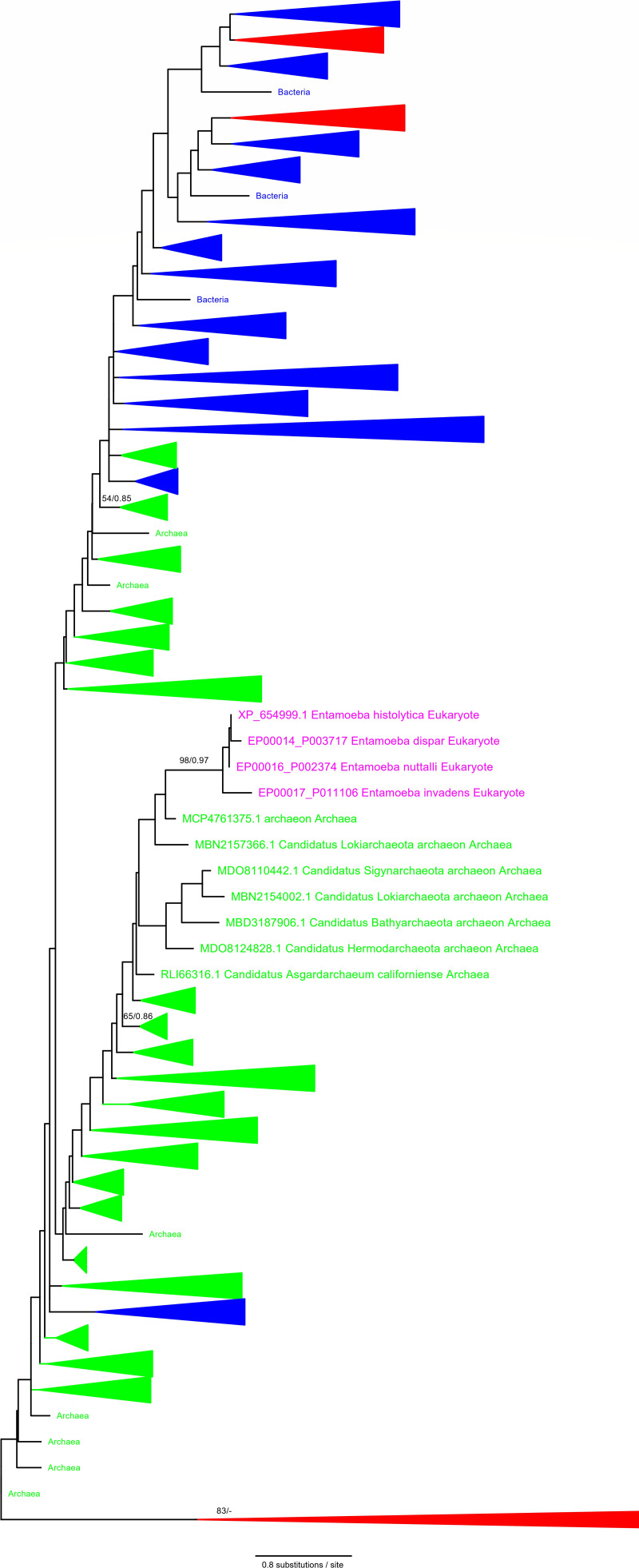
Phylogenetic analysis of EhFADS. Phylogenetic tree of FADS from *E. histolytica* and other organisms. Optimal ML tree inferred was shown from 966 OTUs based on 64 a.a. positions. For each bipartition, the MLBPs are shown at the left of the slash, if greater than 50%, and Bayesian posterior probabilities are shown at right of slash, if greater than 0.8. The color of the letters of OTU indicates the classification of the organism. Red represents eukaryotes; green represents archaea; blue represents bacteria; and purple represents *Entamoeba*. The triangles on the phylogenetic tree are collapsed monophyletic clades, and their colors are those of the majority of organisms in that clade. FADS, flavin adenine dinucleotide synthase; MLBP, maximum likelihood bootstrap value; OTU, operational taxonomic unit.

Phylogenetic analyses of FADS alignment inferred an optimal maximum likelihood (ML) tree shown in Fig. 4 (detailed figure is available in the supporting information; Fig. S1). The results showed that EHI_198800 (putatively EhFADS, hereinafter; enzymatic proof is given below) was in the Archaeal clade, although it was not supported to be statistically significant. All FADS sequences from the genus *Entamoeba* are included in the monophyletic clade with statistical significance of maximum likelihood bootstrap values (MLBPs) of 98% and Bayesian posterior probabilities (BPPs) of 0.97. The eukaryotic-type FADS is clearly phylogenetically distinct from the prokaryotic-type FADS, suggesting that EhFADS was acquired in lateral gene transfer (LGT) by an *Entamoeba* ancestor from an archaeon. Furthermore, we identified several eukaryotic FADS with a prokaryotic origin as shown in red within the blue bacterial clade (Fig. 4). Since most of them belong to the bacterial clade, only *Entamoeba* apparently experienced LGT from archaea.

### Recombinant EhFADS (EHI_198800) showed FAD synthase activity

**Fig 5:**
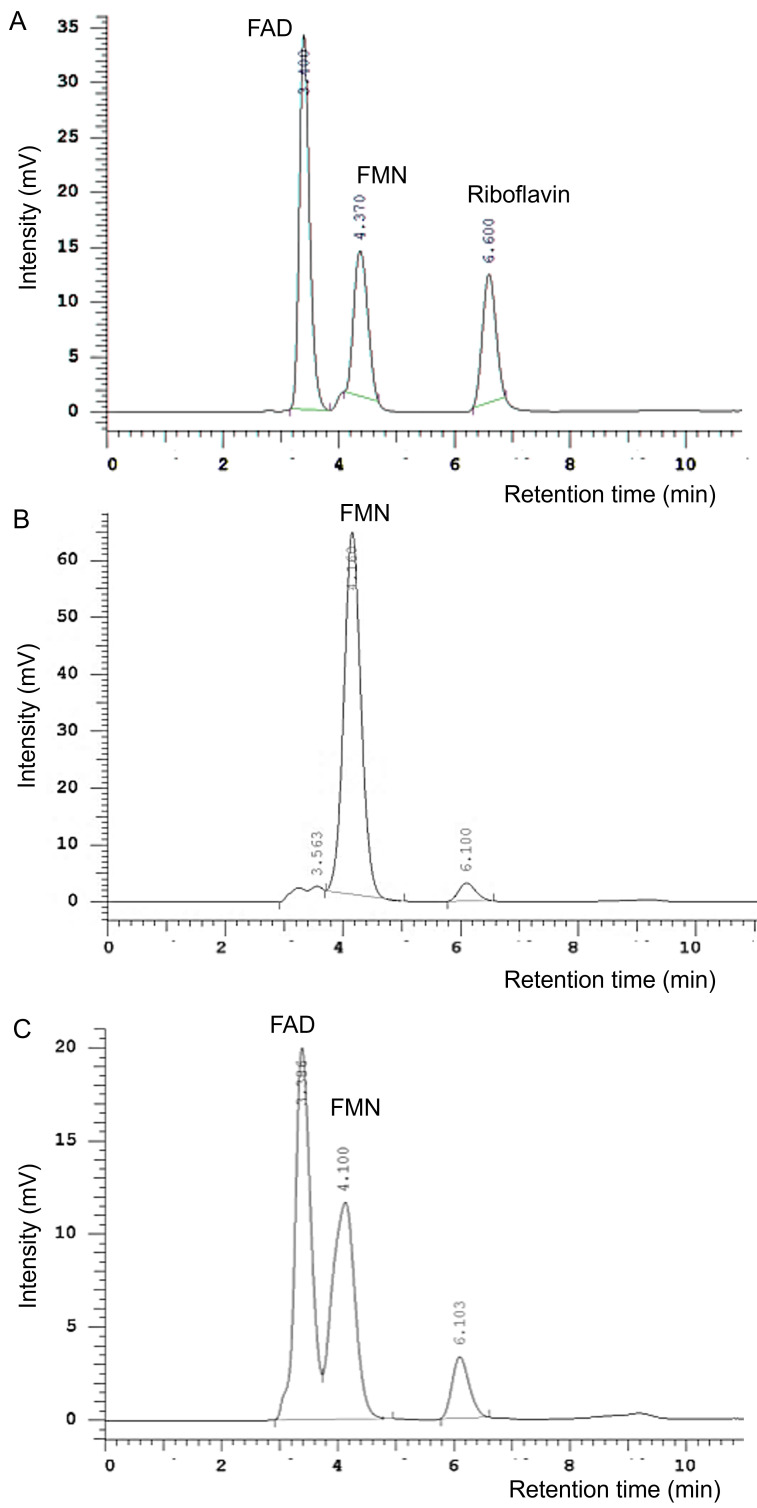
Conversion of FMN to FAD by recombinant EhFADS. (**A**) HPLC analysis of FAD, FMN, and riboflavin standards. (**B** and **C**) FAD formation was monitored by fluorescence detection post-HPLC separation, after incubation with Tris-HCl buffer, pH 7.5, containing FMN, ATP, MgCl_2_, and EhFADS at 35°C for 0 (**B**) and 30 min (**C**). HPLC, high-performance liquid chromatography.

To demonstrate the FAD synthase activity of putative EhFADS (EHI_198800), we produced recombinant protein and evaluated its activity. The recombinant 6xHis-EhFADS was successfully expressed in *Escherichia coli* BL21(DE3) using pCOLDI expression vector and purified to homogeneity. Coomassie brilliant blue staining of sodium dodecyl sulfate-polyacrylamide gel electrophoresis (SDS-PAGE) and immunoblot analysis confirmed the correct size of the protein (Fig. S2). The recombinant protein converted FMN to a compound, in the presence of ATP and magnesium ion at 35°C for 30 min, which showed the retention time of 3.386 min, which agreed well with the FAD standard (3.400), and fluorescence at excitation and emission wavelengths of 450 and 520 nm, respectively (Fig. 5). The formation of this new fluorescence compound was accompanied by the reduction of FMN peak, indicating that FMN is converted into FAD in this reaction.

To determine if pyrophosphate (PPi) is the secondary product of the reaction catalyzed by EhFADS, the production of PPi was monitored using inorganic pyrophosphatase (IPP) as a coupling enzyme and malachite green reagent for phosphate determination. The production of PPi was confirmed (Table S1). PPi was not detected without the addition of the coupling enzyme, IPP, affirming that only pyrophosphate, not phosphate, was generated in this reaction. Taken together, these data confirmed that EHI_198800 is FAD synthase.

### Determination of kinetic parameters of EhFADS

**Fig 6:**
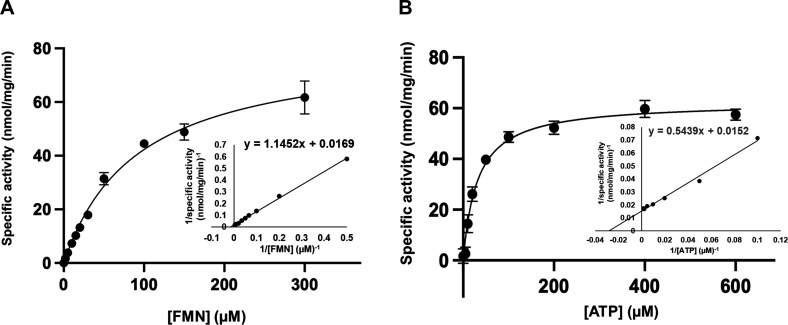
Steady-state kinetics of EhFADS. The specific activity of EhFADS (nanomole per milligram per minute) was measured in the presence of 2–600 μM of FMN and 100-µM ATP (**A**), or 5–600 μM of ATP and 200-µM FMN (**B**) after 30-min incubation at 35°C. The plots shown are representative of three independent experiments (*n* = 3; error bars represent SDs).

The recombinant EhFADS exhibited enzyme dose-dependent activity under the given assay conditions described in the experimental procedures (Fig. S3). The enzyme was active at a wide range of pH 6.0–9.0 with the optimum activity at pH 7.0 (Fig. S3). The plot of EhFADS-specific activity against FMN concentration (5–300 μM) at 100-µM ATP was fit to a Michaelis-Menten curve with an apparent *K*_M_ value of 70.7 ± 7.1 µM and *k*_cat_ value of 1.21 ± 0.12/min (Table 1). EhFADS also exhibited a hyperbolic Michaelis-Menten curve when assayed with a fixed saturating concentration of FMN (200 µM) and varying concentrations of ATP (5–600 µM), with an apparent *K*_M_ value of 43.9 ± 7.9 µM for ATP and *k*_cat_ value of 1.31 ± 0.02/min (Fig. 6B) (Table 1).

**TABLE 1:** Kinetic parameters of EhFADS^*a*^

Substrate	*K*_M_ (μM)	*V*_max_ (nmol/min/mg)	*k*_cat_ (per min)
FMN	70.7 ± 7.1	60.0 ± 5.8	1.21 ± 0.12
ATP	43.9 ± 7.9	64.9 ± 1.1	1.31 ± 0.02

^*a*^ The kinetics of EhFADS were established under steady-state conditions. The fixed concentration of FMN at 200 mM and varied concentrations of ATP ranging from 0 to 600 mM were used to determine the kinetic values for ATP. The fixed concentration of ATP at 100 mM and varied concentrations of FMN ranging from 0 to 300 mM were used to determine the kinetic values for FMN. The values are the average ± SD of three independent experiments.

### Nucleoside donor specificity, metal ion dependence, and reverse reaction of EhFADS

The activity of EhFADS was assessed with various nucleoside donors including CTP, GTP, TTP, and UTP. The enzyme exhibited activity exclusively toward ATP, with no detectable activity observed using other nucleoside donors (Table 2). Like other FADS, EhFADS relies on bivalent metal ions for its catalytic reaction. EhFADS could utilize various bivalent metal ions including Mg^2+^, Co^2+^, Mn^2+^, Ni^2+^, and Zn^2+^ with comparable activity as summarized in Table 3.

**TABLE 2:** Specific activity of EhFADS toward different nucleoside triphosphates^*a*^

Nucleoside triphosphates	Specific activity (nmol/min/mg)
ATP	24.2 ± 3.6
CTP	nd^*b*^
GTP	nd
UTP	nd
TTP	nd

^*a*^ Specific activity was measured in a reaction containing 50-mM FMN, 100-mM ATP/CTP/GTP/UTP, 5-mM MgCl_2_, 50-mM Tris-HCl, pH 7.5, and 2.5-mg protein. The values are the average ± SD of three replicates.

^*b*^ nd, not detected.

**TABLE 3:** Specific activity of EhFADS in the presence of different cations^*a*^

Cations	Specific activity (nmol/min/mg)
None	nd^*b*^
KCl	1.4 ± 0.3
NaCl	nd
LiCl	nd
CaCl_2_	nd
CoCl_2_	21.8 ± 1.4
CuCl_2_	0.8 ± 0.6
MgCl_2_	21.4 ± 1.0
MnCl_2_	26.2 ± 1.3
NiCl_2_	29.6 ± 3.8
ZnCl_2_	20.4 ± 1.4

^*a*^ Specific activity was measured in a reaction containing 50-mM FMN, 100-mM ATP, 5 mM of cations, 50-mM Tris-HCl, pH 7.5, and 2.5-mg protein. The values are average ± SD of three replicates.

^*b*^ nd, not detected.

FADS from humans and some bacteria exhibited reverse activity: utilizing FAD and PPi and producing FMN and ATP (26, 27). Incubation of EhFADS with FAD and PPi, under the given assay conditions, showed the formation of FMN, indicated by fluorescence increase, with the rate of 3.31 ± 0.09 nmol/min/mg, 10 times slower to the forward reaction.

### Effect of *EhFADS* gene silencing on the cellular FAD levels and parasite growth

**Fig 7:**
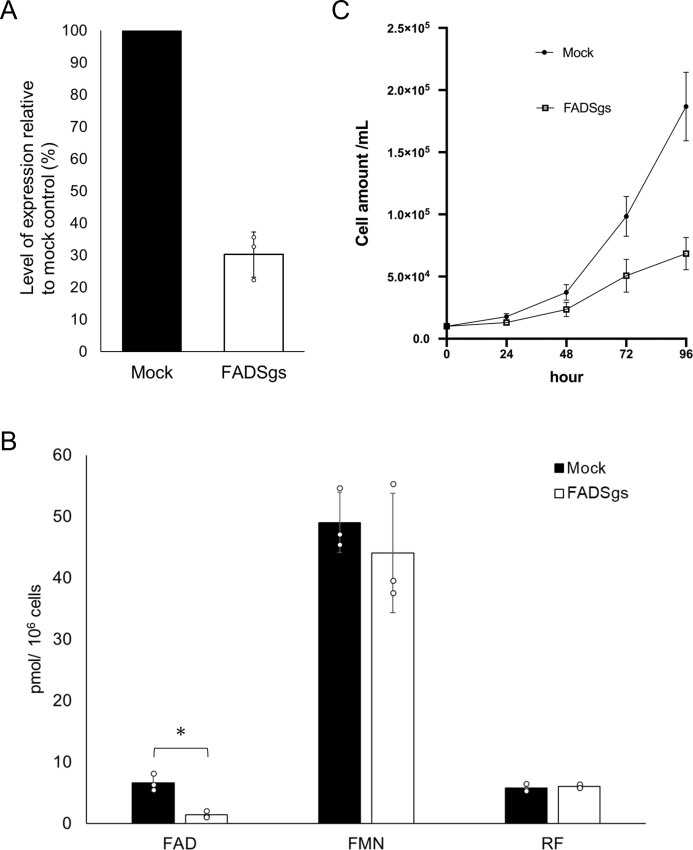
Effects of *EhFADS* gene silencing on growth and flavin levels of *E. histolytica* trophozoites. (**A**) Evaluation of gene expression by quantitative real-time PCR in the *EhFADS* gene silenced strain (*n* = 3; error bars represent SDs). The steady-state levels of transcripts of *EhFADS* and *EhRNA pol II* gene, as a control, were analyzed in *EhFADS* gene silenced and mock control pSAP2G transformants. (**B**) Intracellular amounts of FAD, FMN, and riboflavin in *EhFADS* gs and mock control transfectants. Data shown are the means ± SD of three replicates. The asterisk indicates *P* < 0.05. (**C**) Growth kinetics of *EhFADS* gene silenced (*EhFADS* gs) and control (mock) strains. Approximately 6 × 10^4^ trophozoites in the logarithmic growth phase were inoculated into 6-mL fresh BI-S-33 medium and cultivated at 35.5°C. Amebae were counted every 24 h for 96 h. Data shown are the means ± SD of three independent experiments.

To investigate the physiological importance of EhFADS, repression of *EhFADS* gene expression was performed using antisense small RNA-mediated transcriptional gene silencing (28). *EhFADS* was successfully silenced in *EhFADS* gene silenced (*EhFADS* gs) strain with approximately 70% (69.7 ± 7.1%) reduction of transcript compared to mock pSAP2-gunma (pSAP2G) control, confirmed by quantitative real-time PCR (qRT-PCR) (Fig. 7A). The cellular FAD level significantly decreased to approximately 21% (21.2% ± 4.6%) of the mock control, while FMN showed only a slight (insignificant) reduction and riboflavin remained unchanged (Fig. 7B). *EhFADS* gene silencing led to severe growth defect of the parasite, with the cell population reaching only 36% of the mock control after 96 h of cultivation in normal BI-S-33 medium (Fig. 7C). This suggests that this gene is very important for parasite proliferation.

### Effect of *EhFADS* gene silencing on the activity of flavin-dependent enzymes

**Fig 8:**
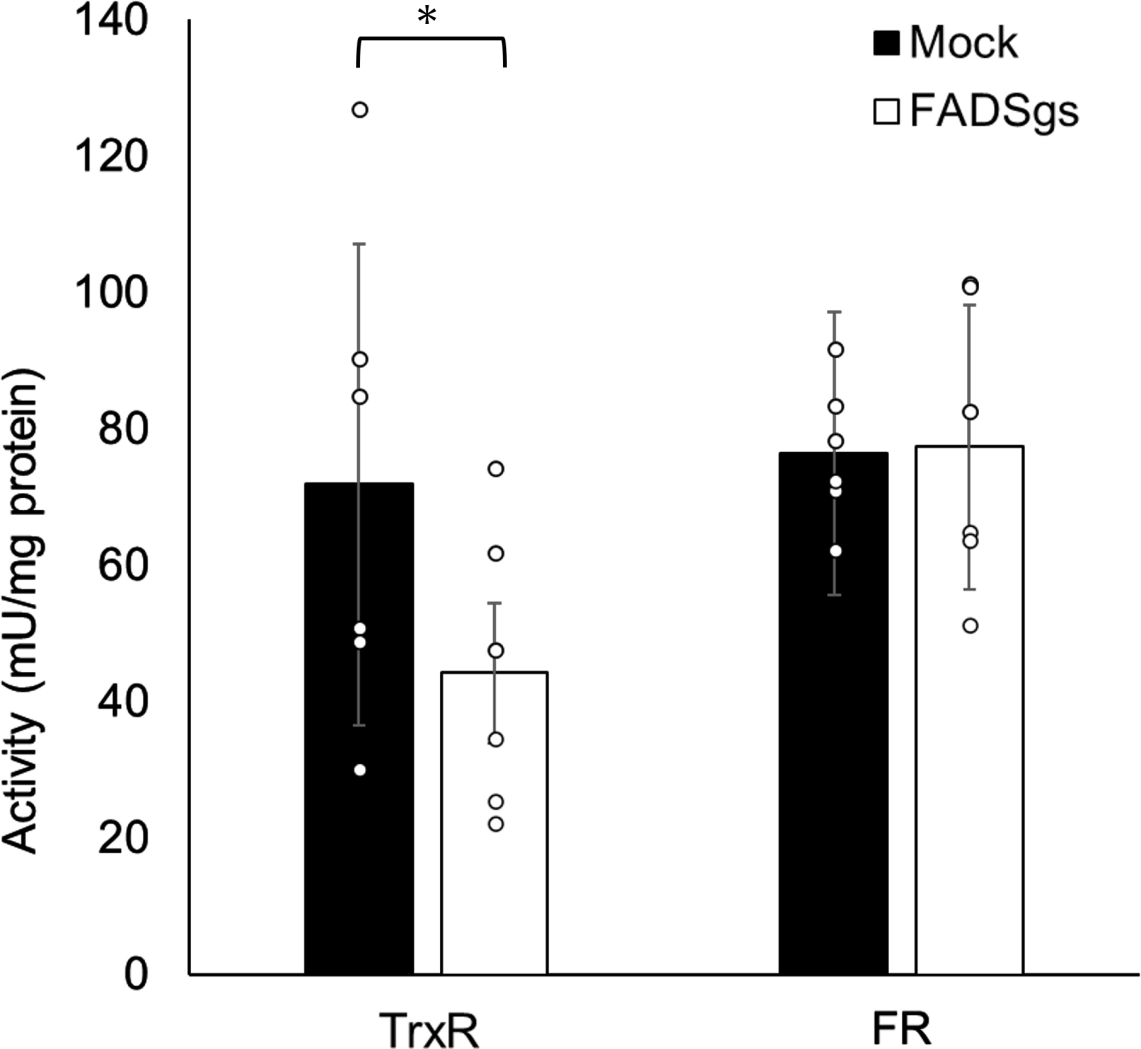
Effects of *EhFADS* gene silencing on intracellular TrxR and FR activity. TrxR (**A**) and FR (**B**) activities were estimated in the lysates from approximately 10^7^ trophozoites of *EhFADS* gene silenced and mock control strains and presented in mU activity of enzyme per milligram protein are shown. One unit is defined as the amount of enzyme that catalyzed the reaction of one micromole substrate per minute under the specified conditions of the assay method. Statistical analysis was performed using paired *t*-tests (three independent experiments, two technical replicates). The asterisk indicates *P* < 0.05. FR, flavin reductase; TrxR, thioredoxin reductase.

To assess the impact of reduced cellular flavin levels on flavin-dependent enzyme activities, we evaluated the activity of TrxR and flavin reductase (FR) in *EhFADS* gs and mock control strains. The activity of TrxR in *EhFADS* gs strain decreased to 61.5% ± 28.9% compared to mock control (*t* = 4.545, *P* = 0.0452), while FR activity remained unchanged (Fig. 8).

### Effect of *EhFADS* gene silencing on metronidazole and auranofin sensitivity

Since TrxR was previously identified as a target of anti-amebic compounds, metronidazole and auranofin, we examined the sensitivity of *EhFADS* gs strain against the compounds. *EhFADS* gs strains were more sensitive to metronidazole (IC_50_ 3.31 ± 3.11 µM) compared to the mock control (8.49 ± 1.52 µM) (Table 4). In contrast, no difference in auranofin sensitivity was observed between the two strains. IC_50_ values against *EhFADS* gs and mock control were 0.93 ± 1.13 µM and 1.13 ± 1.35 µM, respectively.

**TABLE 4:** Metronidazole and auranofin IC_50_ values against EhFADS gs and pSAP2G strains^*a*^

*E. histolytica* strains	IC_50_ (μM)	
	Metronidazole	Auranofin
EhFADS gs	3.31 ± 3.11	0.93 ± 1.13
pSAP2G	8.49 ± 1.52	1.13 ± 1.35

^*a*^ IC_50_ values are the average ± SD of two independent experiments, each with three replicates.

### Effect of *EhFADS* gene silencing on global transcriptome

We performed RNA seq analysis to investigate the effects of *EhFADS* gene silencing on the global gene expression. We identified 47 genes that were differentially expressed more than threefolds with a *Q* value of <0.01. Out of 47 genes, 16 genes (34.1%) were downregulated (Table 5), and 31 genes (65.9%) were upregulated (Table 6). Out of the 16 downregulated genes, 11 genes (68.75%) were with unknown function (hypothetical protein), while the 5 remaining genes were assigned to AIG1 family protein (EHI_072850), competence protein ComEC (EHI_067910), Rab family GTPase (EHI_021480), N-acetylmuraminidase pseudogene (EHI_058470), and phosphatidate cytidylyltransferase activity (EHI_163240). Phosphatidate cytidylyltransferase/CDP diacylglycerol synthetase participates in glycerophospholipid metabolism and phosphatidylinositol signaling by providing CDP-DAG. The gene was also downregulated in *EhG3PDH* gene silenced strain (29) Out of the 31 upregulated genes, 18 genes encoded hypothetical proteins; 4 genes encoded AIG family proteins; and 2 genes were assigned to alkyl sulfatase, while 7 remaining genes were related to ATP binding, protease, and GTPase activity. Among the most upregulated genes were EHI_196760 and EHI_020830, both encoding hypothetical proteins and were upregulated more than 84- and 18-folds, respectively. Interestingly, these genes were two of the most significantly downregulated genes in the cysteine synthase gs strain (30). Despite the reduction in FAD level and the increase in sensitivity to metronidazole, surprisingly, none of FAD- or FMN-dependent genes [such as thioredoxin reductase (EHI_155440) and NADPH:flavin oxidoreductase] and metronidazole-responsive or metronidazole resistance-related genes were affected more than threefold by *EhFADS* gene silencing (19, 31–36). This suggests that there is little or no interaction between *EhFADS* and other genes encoding FAD/FMN-dependent proteins. Instead, the reduction of FAD levels may influence protein activity at the post-translational levels. The transcript level of *EhRFK*, the gene encoding the first step of FAD biosynthesis preceding EhFADS, was also unchanged (data are not shown).

**TABLE 5:** List of the genes that were downregulated >3 fold upon FADS gene silencing

No.	Gene ID	Expression level		Fold change	*Q* value	Gene annotation
		pSAP2G	FADSgs			
1	EHI_179700	76.8	5.2	15.1	6.0E-23	Hypothetical protein
2	EHI_072850	155.1	14.5	10.8	5.5E-23	AIG1 family protein, putative
3	EHI_072740	262.2	40.8	6.4	1.1E-24	Hypothetical protein
4	EHI_012440	8.3	1.6	5.1	8.1E-03	Hypothetical protein
5	EHI_067910	751.6	165.8	4.5	6.0E-12	Competence protein ComEC, putative
6	EHI_010150	18.6	4.0	4.5	2.5E-04	Hypothetical protein
7	EHI_120750	34.3	8.1	4.2	8.4E-04	Hypothetical protein
8	EHI_073060	442.7	106.2	4.2	5.9E-18	Hypothetical protein
9	EHI_129830	181.0	48.4	3.7	2.7E-06	Hypothetical protein
10	EHI_058470	1,080.8	298.4	3.6	3.1E-11	N-acetylmuraminidase pseudogene
11	EHI_180400	23.3	6.3	3.6	3.5E-03	Hypothetical protein
12	EHI_163240	21,940.4	6,173.3	3.6	2.8E-08	Phosphatidate cytidylyltransferase, putative
13	EHI_004930	15.1	4.2	3.5	3.9E-03	Hypothetical protein
14	EHI_158340	44.1	13.6	3.3	9.4E-04	Hypothetical protein
15	EHI_198800	239.9	74.6	3.2	2.4E-12	Cytidylyltransferase, putative
16	EHI_123230	1,321.8	417.6	3.2	3.4E-08	Hypothetical protein, conserved
17	EHI_021480	18.2	5.9	3.1	7.2E-03	Rab family GTPase

**TABLE 6:** List of the genes that were upregulated >3-fold upon FADS gene silencing

No.	Gene ID	Expression level		Fold change	*Q* value	Gene annotation
		pSAP2G	FADSgs			
1	EHI_196760	0.3	41.2	84.8	2.0E-19	Hypothetical protein
2	EHI_020830	1.1	22.7	18.8	1.3E-09	Hypothetical protein
3	EHI_095510	1.8	26.1	13.5	1.4E-10	Hypothetical protein
4	EHI_115720	3.2	41.5	12.2	5.1E-11	Metallo-beta-lactamase superfamily protein
5	EHI_020970	0.7	9.8	11.7	3.1E-04	Hypothetical protein
6	EHI_196780	1.0	14.1	11.6	2.0E-05	Hypothetical protein
7	EHI_035680	5.7	53.0	8.9	6.9E-16	Hydrolase, carbon-nitrogen family
8	EHI_034810	2.6	22.7	8.5	2.2E-07	Hypothetical protein
9	EHI_095310	3.3	26.8	7.9	4.1E-08	Hypothetical protein
10	EHI_165670	3.3	25.7	7.6	6.9E-08	Hypothetical protein
11	EHI_168860	2.2	17.3	7.5	8.9E-06	Hypothetical protein
12	EHI_154760	2.1	16.9	7.4	1.6E-05	Hypothetical protein
13	EHI_072500	2.6	19.6	7.4	7.7E-06	Hypothetical protein
14	EHI_126560	7.2	49.3	6.9	2.3E-07	AIG1 family protein, putative
15	EHI_091670	1.9	13.2	6.8	4.0E-04	Deoxyuridine 5′-triphosphate nucleotidohydrolase domain containing protein
16	EHI_126550	10.6	68.8	6.3	9.5E-11	AIG1 family protein, putative
17	EHI_201610	2.9	16.9	5.6	3.4E-04	Hypothetical protein, conserved
18	EHI_187090	67.0	372.6	5.5	2.0E-22	Rab family GTPase
19	EHI_095100	22.7	126.4	5.5	1.2E-18	Hypothetical protein, conserved
20	EHI_196790	2.8	15.5	5.1	3.6E-04	Hypothetical protein
21	EHI_095480	5.4	26.2	4.7	1.1E-05	Hypothetical protein
22	EHI_178050	9.6	44.2	4.6	1.8E-08	ATP-binding cassette protein, putative
23	EHI_022500	31.0	130.9	4.2	2.2E-12	AIG1 family protein
24	EHI_192130	14.3	56.0	4.0	3.0E-06	Rab family GTPase
25	EHI_078530	65.6	243.1	3.7	1.0E-16	Hypothetical protein
26	EHI_022490	147.8	542.1	3.7	3.5E-19	AIG family protein
27	EHI_169350	10.0	34.1	3.4	7.9E-04	Non-pathogenic pore-forming peptide precursor, putative
28	EHI_080890	11.2	36.1	3.3	2.3E-04	26S protease regulatory subunit, putative
29	EHI_027020	235.7	762.2	3.2	2.2E-16	Hypothetical protein
30	EHI_128900	89.2	289.7	3.2	5.2E-14	Hypothetical protein
31	EHI_171760	123.8	394.1	3.2	7.6E-08	Hypothetical protein

## DISCUSSION

### Discovery of EhFADS, a unique FADS acquired from an archaeal lateral gene transfer

In this study, we have identified and characterized, for the first time, the FAD synthase from *E. histolytica*. EhFADS shows very weak similarity to its counterpart from other organisms including humans, except for archaea. Our phylogenetic analysis (Fig. 4) strongly indicates the archaeal origin of EhFADS, which was likely gained via LGT, a phenomenon well documented in *Entamoebae* (37). Archaea are potential contributors of LGT genes in the *E. histolytica* genome, although archaea-originated genes are not as prevalent in *Entamoeba* as LGT-derived genes from Bacteroidetes and Proteobacteria (38). Interestingly, we found that the archaeal type of FADS is only distributed to the genus of *Entamoeba*, while a eukaryotic type of FADS is present in free-living *Acanthamoeba* and other parasitic protozoa.

Several other archaea-originated genes encoding metabolic enzymes in *Entamoeba* have been reported, including malic enzyme, methionine-γ-lyase (MGL), and the small subunit of glutamate synthase. Malic enzyme is responsible for decarboxylation of malate to pyruvate, while MGL is involved in degradation of sulfur-containing amino acids. *Entamoeba* glutamate synthase small subunit was previously suggested, due to its similarity with archaeal one, to play an enzymatic function without the presence of the large subunit (37). However, later it has been shown that this protein functions as an NADPH-dependent oxidoreductase and may play a role in redox maintenance, L-cysteine/L-cystine homeostasis, iron reduction, and metronidazole activation (32).

### Unique enzymatic features and properties of EhFADS

Despite its archaeal origin of EhFADS, EhFADS exhibits several different properties from archaeal FADS RibL. While RibL can utilize CTP and GTP, in addition to ATP, as nucleotidyl donor with 40% and 10% activity, respectively (24), EhFADS displayed specificity exclusively toward ATP (24). Archaeal FADS contains conserved two cysteine residues which were proposed to be involved in reduced environment sensitivity. Alkylation of these residues leads to a loss of protein activity which cannot be reversed by metal ions addition, suggesting that the two cysteines play a role as a ligand for metal ions. In contrast, no cysteine residues were found in EhFADS (Fig. 2); instead, *Entamoeba* FADS shares different residues, indicating a possible different mechanism in metal ion binding. Determining the protein crystal structure will help to better understand the architecture of the substrate and the metal ion binding.

The kinetic constants of EhFADS (Table 1) are comparable to those of archaeal RibL (*K*_M_ of FMN and ATP, 63 and 25 µM, respectively) and rat liver FADS (91 and 71 µM), but significantly different from human FADS (0.35 and 15.3 µM) (26) and bacteria *Corynebacterium ammoniagenes* FADS (6 and 43 µM) (39).

### Validation of EhFADS as a drug target against amebiasis: functional implications of *EhFADS* gene silencing

We propose that EhFADS is an unexplored rational target for drug development against amebiasis. The justifications for this claim are fivefold: (i) FAD is ubiquitous in life and essential for many biological processes; (ii) as explained above, the unique evolutionary origin and biochemical features of EhFADS; (iii) the essentiality of EhFADS for trophozoite proliferation; (iv) hypersensitivity of *EhFADS* gene silenced strain against metronidazole; and (v) the activity reduction of one important enzyme, TrxR, in *EhFADS* gene silenced strain. The potential of EhFADS as a drug target can be further validated by using an inhibitor of its archaeal protein homolog, RibL. Unfortunately, as of the writing of this paper, no inhibitors for RibL have been identified.

The essentiality of *EhFADS* for trophozoite proliferation has been demonstrated by severe growth defect caused by gene silencing (Fig. 7B). This observation aligns well with the essentiality of FADS in bacteria, such as *C. ammoniagenes*, *Mycobacterium tuberculosis*, and *S. pneumoniae* (40, 41). The reduction in cellular FAD concentrations and the concomitant decrease in enzymatic activity of FAD-dependent TrxR, caused by *EhFADS* gene silencing, may explain these deleterious effects, which is consistent with the previous study on riboflavin deprivation in axenic culture of *E. histolytica* (22). The FAD levels, however, were not determined in the previous study (22) but only predicted to be reduced, while FAD reduction has been directly demonstrated in this study.

A decrease in cellular FAD concentrations may deactivate flavoenzymes, as observed in FADS-silenced *Caenorhabditis elegans*, leading to impaired activity (42). Two of those flavoenzymes with reduced activity were GR and succinate dehydrogenase. In human cell lines, a decrease in FAD concentrations resulting from riboflavin deficiency is associated with reduced activity of GR and reduced glutathione concentration (43). Reduced glutathione plays a vital role in neutralizing free radicals and other reactive oxygen species (ROS), serving as an antioxidant that helps protect proteins, DNA, and other compounds from oxidative damage (43). *E. histolytica* lacks glutathione and glutathione reductase activity (44) and relies on the thioredoxin system as one of the oxidative stress defenses (45). In *EhFADS* gs strain, we observed reduced activity of TrxR by approximately 40% (Fig. 8). Although we did not quantify the endogenous level of the protein, the transcript level remains unchanged, suggesting that the reduced activity is not due to the decreased amount of the protein but may be associated with the low level of cofactor FAD.

### Possible synergy of EhFADS inhibition and existent antiamebic compounds

Another advantage of targeting EhFADS for anti-amebiasis drug discovery is based on the observation that *EhFADS* gene silenced strain is more susceptible to metronidazole, but not auranofin (Table 4). Metronidazole treatment triggered adduct formation of five proteins including TrxR and reduced its enzymatic activity in *E. histolytica* (33). In *EhFADS* gs strains, the inherent reduced activity of TrxR may contribute to its higher sensitivity to metronidazole. Although low TrxR activity and loss of flavin reductase activity were demonstrated to be associated with metronidazole resistance in *Trichomonas vaginalis* (46, 47), these phenotypes have not been recapitulated in *E. histolytica* as no alteration in flavin reductase activity was observed in the *EhFADS* gs strain. A decrease in flavin reductase activity was found to be associated with metronidazole resistance in *Giardia lamblia* and *E. histolytica* (19, 48).

Auranofin inhibits TrxR and its reduction capability in *E. histolytica*, increasing the sensitivity of trophozoites to ROS-mediated killing (34). The overall impact on metabolism by auranofin, however, may differ from that by metronidazole, as several different responses have been reported. Auranofin treatment resulted in an increase in ROS in the trophozoites, while this was not observed in metronidazole treatment at the same concentrations (34). Moreover, it was previously shown that trophozoites which had been adapted to auranofin were more sensitive to metronidazole (49). Although TrxR was assumed to be the primary target of auranofin in *E. histolytica*, the mode of action is not completely understood. It was in fact suggested that EhTrxR does not play a central role in the response and adaptation (49).

### Transcriptomic changes associated with *EhFADS* gene silencing indicate possible correlation with sulfolipid metabolism

We investigated global changes of gene expression profile caused by *EhFADS* gene silencing. It was counterintuitive that the transcript levels of genes encoding flavoproteins were not significantly affected more than threefold, despite the fact that *EhFADS* gene silencing severely affected the cellular FAD levels. These data indicate that gene expression of enzymes that require cofactors such as FAD is not regulated at transcriptional levels by the activities of these enzymes or the concentrations of their metabolites. The lack of regulatory cross-talk between FAD synthesis and gene expression of flavoproteins was also noted in *C. elegans*, where most proteins affected by *FADS* gene knockdown were not flavoproteins (42).

Among the genes that were highly expressed in the parental strain and affected by *EhFADS* gene silencing, a gene encoding phosphatidate cytidylyltransferase or CDP-DAG synthetase (cds) was downregulated 3.6-fold in the *EhFADS* gs strain. CDP-DAG serves as the central intermediate in the synthetic pathways of phosphatidylinositol (PI), glycosylphosphatidylinositol (GPI), phosphatidylglycerol, cardiolipin, and phosphatidylserine (50). The *cds* gene is apparently essential in *Toxoplasma gondii* and *Trypanosoma brucei*. It was shown that *cds* gene knockout resulted in low PI and GPI production and severe growth (51, 52).

It may be also worth noting that a gene encoding alkyl sulfatase (SF) (*EhSF5*, EHI_115720, annotated as metallo-beta-lactamase superfamily protein in AmoebaDB) was upregulated 12-fold in the *EhFADS* gs strain. SF is involved in sulfolipid metabolism. *E. histolytica* has five genes encoding SF (EHI_095100, EHI_198980, EHI_086530, EHI_107070, and EHI_115720). All five SFs show highest similarity to Zn-dependent alkylsulfatases from *Pseudomonas aeruginosa* (32.0%–40.1%) (53). It was demonstrated that knockdown of *EhSF5* resulted in an altered sulfolipid profile, and more specifically, accumulation of a few sulfolipid species, SL-II-IV. Although causal connection between *EhFADS* gene silencing and upregulation of *SF5* remains elusive, these data indicate that FAD metabolism may regulate sulfolipid metabolism and play a role in the parasite proliferation and differentiation.

In conclusion, we identified an evolutionary unique key enzyme, archaeal-type FADS for FAD biosynthesis from *E. histolytica* for the first time. The unique characteristics and essentiality of EhFADS underscore its potential as a rational drug target against *E. histolytica* infections.

## MATERIALS AND METHODS

### Organism and chemicals

Trophozoites of *E. histolytica* HM-1: IMSS cl six and G3 (28) were grown axenically at 35.5°C in Diamond’s BI-S-33 medium as described previously (54). Trophozoites in logarithmic growth phase were used for all experiments. *Escherichia coli* BL21(DE3) strain was purchased from Invitrogen (Carlsbad, CA, USA). Ni^2−^-NTA agarose was purchased from Novagen (Darmstadt, Germany). Lipofectamine and geneticin (G418) were purchased from Invitrogen. FMN, FAD, and all other chemicals of analytical grade were purchased from Sigma-Aldrich (Tokyo, Japan) unless otherwise stated.

### Phylogenetic analysis

We retrieved prokaryotic protein sequences similar to EhFADS by BLASTP search against the National Center for Biotechnology Information (NCBI) protein database using EhFADS (XP_654999.1) as a query. We subsequenty used CDHIT v.4.8.1 (55, 56) against prokaryotic sequences obtained from BLASTP search as the default setting. For eukaryotic sequences, we conducted a BLASTP search against the Eukprot v.3 (https://evocellbio.com/eukprot/) protein database using EhFADS as a query. We assembled prokaryotic and eukaryotic sequences and aligned by MAFFT v.7.520 (57) with the L-INS-i algorithm, followed by manual correction and exclusion of ambiguously aligned positions. The final alignment contained 966 operational taxonomic units with 64 amino acid positions.

The processed alignments were then subjected to the ML method by IQ-TREE v.2.2.0 (58) with the LG + I + I + R10 model, which is the model selected automatically by the ModelFinder (59) built into IQ-TREE. The robustness of the ML phylogenetic tree was evaluated with a non-parametric ML bootstrap analysis (100 replicates). We also conducted Bayesian phylogenetic analysis with the LG + CAT model using Phylobayes mpi v.1.8a (60, 61). In this analysis, two MCMC runs for 100,000 cycles with “burn-in” of 10,000. The consensus tree with branch lengths and BPPs were calculated from the remaining trees. The phylogenetic tree inferred is a maximum likelihood phylogenetic tree, supported by MLBPs and BPPs.

### Cloning and expression of recombinant protein

*EhFADS* (EHI_198800) open reading frame was amplified by PCR from cDNA using primer sets 5′-AC**GGATCC**ATGAAAGGAAAAACAAAAC-3′ as a forward primer and 5′-CG**AAGCTT**TTATTGTATTTTGTTATTTAC-3′ as a reverse primer with the bold letters indicating the restriction sites of BamHI and HindIII, respectively. The digested and purified PCR product was inserted into BamHI and HindII double digested pCOLD1 histidine-tag vector (Takara) containing 6× histidines at the N-terminal to produce recombinant pCOLD1-EhFADS. The sequence of the plasmid was confirmed by Sanger sequencing.

The expression plasmid was introduced into *E. coli* BL21(DE3) by heat shock. The transformant was then cultured in Luria-Bertani (LB) medium containing ampicillin. Protein expression was induced by the addition of 0.5-mM IPTG when the OD_600_ value reached 0.5–0.7 and was further incubated at 15°C for 24 h. The bacterial pellet, collected by centrifugation at 7,500 rpm for 20 min, was resuspended and lysed by incubation with lysis buffer (Tris-HCl, pH 8, NaCl, Triton X-100 0.1%, 1-mM phenylmethylsulfonyl fluoride (PMSF), and 100-µg/mL lysozyme) on ice for 30 min, followed by sonication.

### Purification of recombinant EhFADS

A 100-mL overnight culture of *E. coli* BL21(DE3) harboring pCOLD1-EhFADS was used to inoculate 1-L fresh LB medium containing ampicillin. The protein expression was induced as described above. After incubation at 15°C for 24 h, the pellet cell was collected by centrifugation, washed with cold phosphate-buffered saline (PBS) pH 7.4 and lysed in 50 mL of lysis buffer followed by sonication, as above. The total lysate was centrifuged at 13,000 rpm for 20 min at 4°C to separate the soluble and insoluble fractions. The Nickel-NTA beads (4 mL of 50% slurry) were added to the soluble fraction to allow the recombinant protein to bind to the beads. After extensive washing with a series of increasing (up to 50 mM) concentrations of imidazole, the bound protein was eluted with 10 mL of elution buffer (Tris-HCl, pH 8, NaCl, 1-mM PMSF) containing 200-mM imidazole. The purity of eluted recombinant EhFADS was confirmed by SDS-PAGE, followed by Coomassie brilliant blue staining. The eluted fraction was subjected to repeated cycles of filtration with Amicon 10K to remove the imidazole. The purified protein was kept in 50% glycerol at −80°C until further use.

### FADS assay

The assay of FADS activity was performed according to the protocol previously described (62) with some modifications. Briefly, a 200-µL reaction mixture of 50-mM Tris-HCl, pH 7.5, containing 1-µM FMN, 50-µM ATP, 5-mM MgCl_2_, and recombinant FADS was incubated at 37°C. At 30 min of incubation, 100 µL of cold perchloric acid was added to the reaction mixture to stop the reaction. After vortexing and centrifugation at 13,000 rpm for 5 min at 4°C, the supernatant was transferred into a new microcentrifuge tube, neutralized by the addition of 50 µL of neutralization buffer (5-M KOH/2-M KH_2_PO_4_), vortexed again, and centrifuged. The supernatant was subjected to high-performance liquid chromatography (HPLC) analysis to monitor riboflavin, FMN, and FAD. A reaction without recombinant protein was used as a control.

A Hitachi HPLC Chromaster (Tokyo, Japan) equipped with a Chromaster 5440 fluorescence detector set at 450 nm for excitation and 520 nm for emission, and a YMC C-18 chromatographic column (250 × 4.6 mm, 5-µm particle size) set at 40°C was used for flavin analysis. The samples were kept at 10°C in the autosampler. A mobile phase consisting of 10-mM phosphate buffer, pH 6, and methanol with a gradient elution of 85:15–65:35 vol/vol for 10 min, followed by 65:35–85:15 for 5 min at a flow rate 1 mL/min was used for separating flavins.

Pyrophosphate produced by FADS was monitored with inorganic phosphate produced by pyrophosphatase (IPP) in a coupled assay, using malachite green assay. In brief, a 100-µL reaction mixture of 50-mM Tris-HCl, pH 7.5, containing 50-µM FMN, 200-µM ATP, 5-mM MgCl_2_, 2.5-µg protein, and 1 unit of IPP was incubated at 37°C for 30 min. Approximately 25 µL of the reaction was then aliquoted into a clear 96-well plate, and 100 µL of malachite green reagent (Cell Signal, Japan) was added. The absorbance was read at 630 nm using SpectraMax Paradigm (Molecular Devices, California, USA) spectrofluorometer after incubation at room temperature for 20 min. The reaction without protein was used as a control.

### Determination of EhFADS kinetic parameters

The FAD synthase activity was measured by monitoring changes in the fluorescence profiles of FMN and FAD (62). In brief, a 100-µL reaction mixture of 50-mM Tris-HCl, pH 7.5, containing 50-µM FMN, 100-µM ATP, 5-mM MgCl_2_, and 2.5 µg of protein was incubated at 35°C. Fluorescence changes at excitation at 450 nm and emission at 520 nm were monitored on a SpectraMax Paradigm (Molecular Devices) spectrofluorometer. In each experiment, FMN and FAD fluorescence were calibrated by using standard solutions whose concentrations were calculated by using ε450 of 12.2/mM/cm for FMN and 11.3/mM/cm for FAD.

The rate of FAD synthesis (nmol FAD/min/mg protein) was calculated from the rate of fluorescence decrease, measured as the tangent to the initial part of the experimental curve by applying the following equation:


$$v_{0}=\left[(\Delta F450/520/\Delta \varphi 450/520)\times V_{f}\right]/(t\times m),$$

where ∆*F* is expressed in fluorescence arbitrary units; ∆φ = φFMN − φFAD is expressed as per micromolar; *V*_f_ is total volume reaction expressed in milliliter; *t* is time expressed in minute; and *m* is the mass of protein in milligram.

Various amounts of protein ranging from 0.1 to 3.2 µg were used to ensure enzyme dose dependence. Various concentrations of FMN ranging from 2 to 600 µM with a constant 100-µM ATP, or various concentrations of ATP ranging from 5 to 1,000 µM with a constant 200-µM FMN were used to determine *K*_M_ and *v*_max_. The enzyme activity was fit to Michaelis-Menten equation generated by using GraphPad Prism (GraphPad Software Inc., San Diego, USA).

### Generation of *EhFADS* gene silenced strains

The plasmid pSAP2G (63) was used for construction of an epigenetic silencing of FADS cell lines. A 402-bp fragment of the *EhFADS* coding region was amplified using the following primer set: 5′-CATGGCCTTGAGGTTATGATTGCTGGTACA-3′ and 5′-ACGAGCTCTCTCCGTATTTGAGAAGATGATTG-3′. The PCR product was cloned into StuI and SacI restriction sites of pSAP2G. The final construct was confirmed by sequencing. *E. histolytica* G3 strain trophozoites were transfected with the *EhFADS* gene silencing plasmid or the control mock pSAP2G plasmid by lipofection as described previously (64). Transfectants were initially selected by cultivating trophozoites with 1-µg/mL G418. G418 was gradually increased to 10 µg/mL in the next 2–3 weeks. Stable transfectants were maintained under this level of G418.

### Reverse transcriptase PCR

RNA was isolated from 1 × 10^6^ trophozoites using Trizol reagent (Ambion; Life Technologies, Grand Islands, NY, USA) according to the manufacturer’s instructions. DNase treatment was performed using DNase I (Invitrogen) to exclude genomic DNA. cDNA was synthesized using SuperScript III First-Strand Synthesis System (Invitrogen) according to the manufacturer’s instruction. The cDNA product was diluted 10-fold and PCR-amplified using Ex-Taq polymerase (Takara) with the following primer set: 5′-TTGCACGTGACAGTGTTGTT-3′ and 5′-GGCTCATACCAATTCCCATT-3′. RNA levels were normalized to the reference gene, RNA polymerase II gene (EHI_056690), quantified using a primer set: 5′-TGCGGGATATAGACAACCTCA-3′ and 5′-TTCCTACTGATGCAGCTTCAAA-3′.

### qRT-PCR

The relative mRNA level of *FADS* was measured by qRT-PCR using primer sets 5′-TTGCACGTGACAGTGTTGTT-3′ and 5′-GGCTCATACCAATTCCCATT-3′. The PCR reaction contained 10-µL 2× Fast SYBR Green Master Mix (Applied Biosystems, Foster City, CA, USA), 0.6 µL each of 10-µM primers, 5-µL diluted cDNA, and nuclease-free water in a total volume of 20 µL. PCR was performed using StepOne Plus Real-Time PCR System (Applied Biosystems) with the following cycling conditions: 95°C for 20 s, followed by 40 cycles 95°C for 3 s and 60°C for 30 s. All reactions were carried out in triplicate, including control without cDNA. The steady-state of mRNA level of *FADS* was determined by the double delta Ct value method with RNA pol II used as a reference gene.

### Growth monitoring of *EhFADS* gene silenced strains

The growth of *EhFADS* gene silenced and mock pSAP2-Gunma strains was monitored for 4 days. A total of 60,000 trophozoites for each strain were used to inoculate 6-mL fresh BI-S-33 medium supplemented with 10 µg/mL of G418. The number of trophozoites was counted every 24 h using a hemhocytometer. Three replicates from different tubes were used for the observation.

### Flavin quantitation in cell lysates

A total of 1 × 10^6^ cells were pelleted by centrifugation at 500 × *g* for 5 min. The pellet was washed twice with cold PBS, pH 7.4, and mixed with 200 µL of cold perchloric acid. The mixture was vortexed and centrifuged at 16,000 × *g* at 4°C for 5 min. The supernatant was transferred to a new tube and neutralized by the addition of 100-µL neutralization buffer, vortexed, and centrifuged as above. The supernatant was subjected to HPLC analysis for flavin determination as described above.

### Enzyme assay in cell lysate

Approximately 10^6^ trophozoites of 48-h old culture were lysed in 50 µL of ameba lysis buffer of 50-mM Tris-HCl, pH 7.5, 150-mM NaCl, containing 0.2% Triton X-100, 0.5-mg/mL E64, and protease inhibitor cocktail. The mixture was vortexed, incubated on ice for 30 min, and sonicated. The lysate and the pellet were separated by centrifugation at 13,000 rpm at 4°C for 10 min. The thioredoxin-reducing (TrxR) activity of cell lysates was indirectly measured by determining the reduction of 5,5′-dithio-bis-(2-nitrobenzoic acid) in the presence of NADPH as described previously (46). Flavin reductase activity was determined by measuring the consumption of NADPH (0.2 mM) after the addition of riboflavin (10 mM), by monitoring absorption at a wavelength of 340 nm. Cell lysates of 25-µg protein equivalent were used for the assays.

### RNA-seq analysis

Approximately 10^6^ of *E. histolytica* trophozoites of *EhFADS* gene silenced and pSAP2G mock transfected strains were harvested at logarithmic growth phase. Total RNA was purified using TRIZOL reagent (Thermo Fisher, Waltham, MA, USA) according to manufacturer’s instruction. Total RNA samples from three biological replicates were sent for RNA sequencing (Macrogen, Kyoto, Japan). RNA-seq libraries were generated by using TruSeq stranded mRNA kit. PolyA plus RNA purification, RNA fragmentation, random hexamer primed cDNA synthesis, linker ligation, PCR amplification, and gel purification were conducted by Macrogen. The libraries were then subjected to 100-nt paired-end sequencing using an Illumina NovaSeq 6000 (Illumina, San Diego, CA, USA). Raw image files were processed using the Illumina Real Time Analysis software. Data analysis was conducted by Tohoku Kagaku (Iwate, Japan). Briefly, adaptor sequences were removed from all reads, and low-quality reads were removed. Obtained high-quality reads were mapped to *E. histolytica* HM-1:IMSS template genome at AmoebaDB, then fragments per kilobase of exon per million mapped fragments were calculated (Tohoku Kagaku).

### Determination of metronidazole and auranofin IC_50_

Approximately 1 × 10^4^ trophozoites of *E. histolytica EhFADS* gs and pSAP2G control strains were suspended in 200 µL of BI-S-33 medium and dispensed into each well of a 96-well plate and incubated at 35.5°C for 2 h. The medium was then removed and replaced with 200 µL of fresh BI-S-33 medium that contained various concentrations of either metronidazole or auranofin (0.2–25 µM). The plate was incubated under anaerobic conditions at 35.5°C for 24 h for auranofin-treated trophozoites and 48 h for metronidazole-treated trophozoites. After the medium was removed, 100 µL of pre-warmed Opti-MEM I (Life Technologies) containing 1/10 volume of WST-1 (Roche, Mannheim, Germany) was added. The viability of trophozoites was estimated by measuring absorbance at 450 nm by SpectraMax Paradigm (Molecular Devices). Cells cultivated with only 0.5% dimethyl sulfoxide (DMSO) were used as negative control. Each assay was performed in triplicate. IC_50_ values were calculated by least squares curve fitting of the dose inhibition curves using GraphPad Prism.

## Data Availability

The data sets generated and/or analyzed during the current study are available from the corresponding author on reasonable request.

## SUPPLEMENTAL MATERIAL

Supplemental material msphere.00347-24-s0001.pdf Supplemental figures and table.

ASM does not own the copyrights to Supplemental Material that may be linked to, or accessed through, an article. The authors have granted ASM a non-exclusive, world-wide license to publish the Supplemental Material files. Please contact the corresponding author directly for reuse.
