# Supplementary material for: Identification and characterization of archaeal-type FAD synthase as a novel tractable drug target from the parasitic protozoa Entamoeba histolytica
Source: mSphere. 2024 Aug 27;9(9):e00347-24. doi: 10.1128/msphere.00347-24 (PMC11423594; doi:10.1128/msphere.00347-24)
Supplement: Supplemental material — Supplemental figures and table. [file msphere.00347-24-s0001.pdf]

Supplemental materials

Identification and characterization of archaeal-type FAD synthase as a novel tractable drug target from the parasitic protozoa *Entamoeba histolytica*

Dewi Wulansari, Ghulam Jeelani, Euki Yazaki, Tomoyoshi Nozaki

List of supplemental materials:

Table S1. Absorbance value of pyrophosphate identification by malachite green assay

Figure S1. Phylogenetic analysis of EhFADS

Figure S2. Expression and purification of recombinant EhFADS in *E. coli*

Figure S3. Dose and pH dependent of recombinant EhFADS activity

Table S1. Absorbance value of pyrophosphate identification by malachite green assay

Assay reaction	Absorbance (arbitrary unit)
Complete reaction	0.77 ± 0.01
Complete reaction minus EhFADS	0.27 ± 0.01
Complete reaction minus IPP	0.22 ± 0.003

Complete reaction contains of 50 µM FMN, 200 µM ATP, 5 mM $MgCl_2$, 50 mM Tris HCl pH 7.5, 2.5 µg protein EhFADS, and IPP (inorganic pyrophosphatase). Absorbance is the average of three replicates, ± SD.

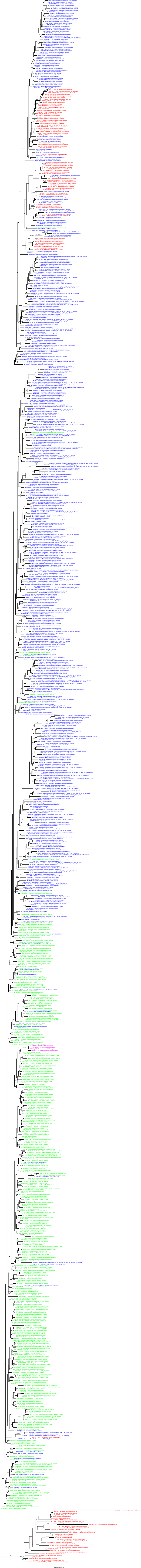

Figure S1. Phylogenetic analysis of EhFADS. Phylogenetic tree of flavin adenine dinucleotide synthase (FADS) from *E. histolytica* and other organisms. Optimal ML tree inferred was shown from 966 OTUs based on 64 a.a. positions. For each bipartition, the ML bootstrap support values (MLBPs) are shown at left of slash, if greater than 50%, and Bayesian posterior probabilities (BPPs) are shown at right of slash, if greater than 0.8. The color of the letters of OTU indicates the classification of the organism. Red represents eukaryotes, green represents archaea, blue represents bacteria, and purple represents *Entamoeba*. The triangles on the phylogenetic tree are collapsed monophyletic clades, and their colors are those of the majority organisms in that clade.

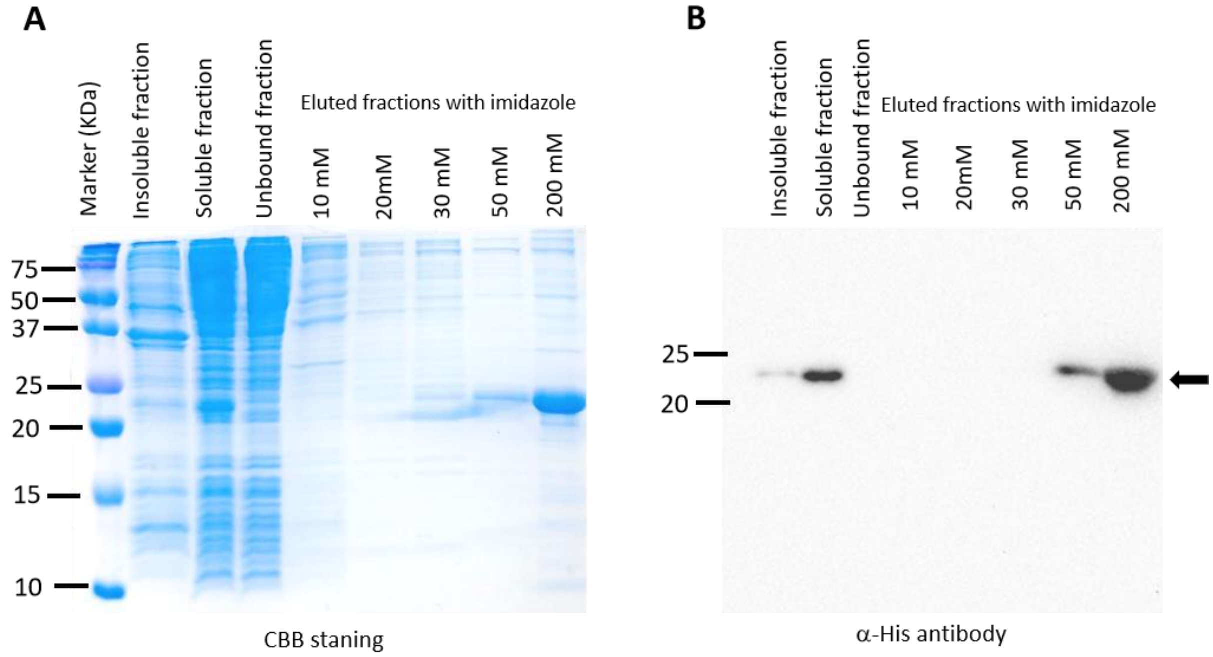

Figure S2. Expression and purification of recombinant EhFADS in *E. coli*. Protein samples at each step of purification were subjected to 20% SDS-PAGE. The gel was then stained with Coomassie Brilliant Blue (A) or proceeded to immunoblot analysis using anti-His-tag antibody (B). Arrow indicates the recombinant His_6-EhFADS (23 KDa).

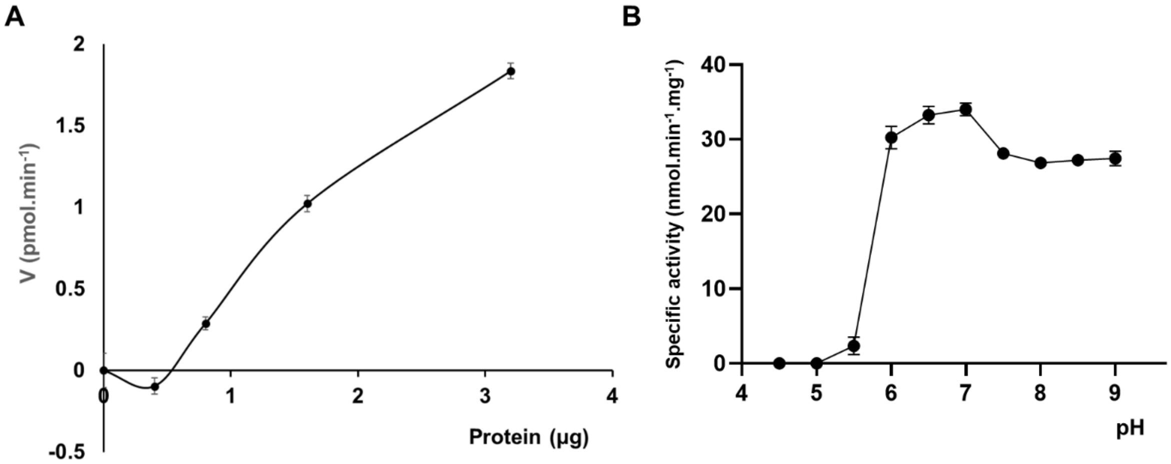

Figure S3. Dose and pH dependent of recombinant EhFADS activity. (A) Plot of EhFADS activity using various amount of protein (0.2, 0.4, 0.8, 1.6, and 3.2 µg) per reaction. The activity was measured in the present of 50 µM FMN, 100 µM ATP, 5 mM $MgCl_2$, and 50 mM Tris HCl pH 7.5. n=3, error bars are SD. (B) EhFADS activity was determined over a range of pH's using the following buffer: pH 4.5 to 5.5 (50 mM acetate), pH 6 to 7 (50 mM MOPS), and pH 7.5 to 9 (50 mM Tris HCl). The specific activity was measured in the present of 50 µM FMN, 100 µM ATP, 5 mM $MgCl_2$, and 2.5 µg protein. n=3, error bars are SD.
